# An Aerosol Extinction Coefficient Retrieval Method and Characteristics Analysis of Landscape Images

**DOI:** 10.3390/s21217282

**Published:** 2021-11-01

**Authors:** Dukhyeon Kim, Youngmin Noh

**Affiliations:** 1 School of Basic Science, Hanbat National University, Daejeon 34158, Korea; 2 Department of Environmental Engineering, Pukyong National University, Busan 48613, Korea; nym@pknu.ac.kr

**Keywords:** extinction coefficients, ångström exponent, camera-rgb, aerosol, effective wavelength, PM_2.5_

## Abstract

Images based on RGB pixel values were used to measure the extinction coefficient of aerosols suspended in an atmospheric state. The pixel values of the object-image depend on the target-object reflection ratio, reflection direction, object type, distances, illumination intensity, atmospheric particle extinction coefficient, and scattering angle between the sun and the optical axes of the camera, among others. Therefore, the imaged intensity cannot directly provide information on the aerosol concentration or aerosol extinction coefficient. This study proposes simple methods to solve this problem, which yield reasonable extinction coefficients at the three effective RGB wavelengths. Aerosol size information was analogized using the RGB Ångström exponent measured at the three wavelengths for clean, dusty, rainy, Asian dust storm, and foggy days. Additionally, long-term measurements over four months showed reasonable values compared with existing PM_2.5_ measurements and the proposed method yields useful results.

## 1. Introduction

Numerous studies have attempted to obtain aerosol information because it is important for human health, traffic and airport safety, and climate change [1,2]. Many systems and methods exist for measuring aerosols, one of which measures the aerosol optical scattering cross-section (area), whereas another measures the mass density (volume) of the dried aerosol.

The total surface area of an aerosol within a given volume is important because it also determines the chemical reaction speed and reaction characteristics, as a greater area has a higher probability of molecule-to-molecule contact. When aerosol particles are inhaled, a high interaction probability increases chemical reactions with other elements that exist at the boundary of the aerosol surface, such as water vapor and other molecules in the human body. The extinction coefficient can be obtained by integrating the product of the extinction efficiency and the surface area distribution. Therefore, the extinction coefficient represents the area of the particle per unit volume.

In contrast, PM_10_, PM_2.5_, PM_1.0_, and TSP (Total Suspended Particles) measure the total integrated mass of dried aerosol within a unit atmospheric volume, providing parameters other than the extinction coefficient. Therefore, we must measure both the total aerosol volume (PM_10_, PM_2.5_, PM_1.0_, and TSP) and aerosol surface with independent equipment and methods, despite the connection between these two parameters based on size distribution information.

Normally, optical methods measure the aerosol cross-section (area) using transmittance or scattering methods, referred to as LiDAR (Light Detection and Ranging), OPC (Optical Particle Counter), visibility system, and nephelometer. Most of these point measurement systems measure small volume and require time to obtain a high signal-to-noise ratio (SNR) at the expense of the temporal resolution [3]. It is general that point detection systems require long term average to cover wide spatial volume. Very high temporal resolution aerosol information is sometimes required to predict or obtain real-time information to prepare for meteorological events, such as fog, as it sometimes forms and disappears rapidly within 30 min [4,5,6]. This information may be necessary as these events sometimes occur in an air-suspended wet-aerosol state, where aerosols include the water absorbed on their surface.

Many studies have attempted to measure visibility using landscape images [7,8,9,10]; however, to the best of our knowledge, no research group has attempted to directly measure aerosol scattering characteristics using landscape images. Studies have attempted to measure the AOD (Aerosol Optical Depth) from sky images using RGB information from the sky [11,12,13,14]. Some studies have measured the aerosol extinction using the changes in the intensity in the captured images for the well-known black–white reference target image by assuming 1st order sunlight scattering and samples deposited on filters [15,16,17,18]. However, for a foggy or high-density aerosol, we cannot assume a 1st scattering approximation, and we cannot use target objects and a sampler for real-time measurements. All sky images contain scattering information for the particles distributed from the bottom to the top of the atmosphere (TOA); furthermore, we can retrieve the aerosol optical depth from the bottom to the TOA [19]. In this case, we should solve the full radiative transfer equations [20] for a given complex cloud distribution and height, as well as for an assumed aerosol vertical distribution for every minute of the day. For this reason, the AOD extraction method using the sky image has a limit in the number data that can be obtained. Other studies have measured the AOD using the same types of images but measured by satellites using the same principle [21,22]. The target reflection scattering characteristic effectively influences the retrieval of the AOD. These AODs are important parameters in climate and weather studies, but most reports focus on the aerosols long vertical direction. The other research groups have used image quality variations as visibility levels [23,24,25,26,27].

To the best of our knowledge, no studies have measured the aerosol extinction coefficients at RGB wavelengths using arbitrary landscape images because a commercial camera measures the image pixel values; these values depend on many parameters, such as the aerosol extinction coefficient, scattering characteristics and object position, position and direction of the sun and camera, atmospheric cloud conditions, and other parameters (such as the sensitivity and transmittance of the imaging optics in the measurement system). In this study, we propose the retrieval of the extinction coefficients using a commercial camera and verify this method under various atmospheric conditions.

The remainder of the paper is organized as follows. In Section 2, we defined RGB effective wavelengths and basic theory in retrieving extinction coefficients. To check basic theory and optimize experimental conditions, we calculated the extinction coefficients for the different experimental condition such as the distances of sky, objects, direction of objects, direction of sun, and the uncertainty of objects reflectivity and particle scattering coefficients. In Section 3, we calculated extinction coefficients at the three RGB effective wavelengths for different weather conditions using the optimized experimental parameters given in Section 2. Finally, we discuss the results and make our conclusions.

## 2. Methodology and Dependence on Experimental Conditions

### 2.1. Theory and Definition of Effective Wavelengths

Imaged pixel values contain information on the target reflectance, atmospheric transmission or extinction-scattering-absorption efficiency, and atmospheric light illumination, among others. Although various information is included in the pixel value, the imaged intensity $I\left(R_{i}\right)$ incident for a given pixel can be described as follows [17,28,29]:

(1) $$\left(R_{i}\right)=C_{1}e^{-\alpha R_{i}}+C_{2}\left(1-e^{-\alpha R_{i}}\right)=(C_{1}-C_{2})e^{-\alpha R_{i}}+C_{2}$$

where ($R_{i}$) is the distance from the target object i to the camera, $C_{1}$ depends on the radiance of the target object, α is the average extinction coefficient between the object and the camera, and $C_{2}$ is the atmospheric scattering radiance scattered by particles from the sun and multiply scattered light. Here, $C_{1}$ depends only on scattering target characteristics, such as reflectance and the direction angle between the sun and camera; however, its value does not depend on distance. The first term of Equation (1) describes the intensity scattered from the target, which decreases exponentially with an increasing target distance owing to atmospheric extinction effects. The second term on the right-hand side of Equation (1) derives from light scattered by atmospheric particles and air, depending on the illumination light, which is composed of direct sunlight and multiply scattered almost isotropic light along the line from the camera to the target. The atmospheric scattering light intensity, which is related to the amount of light scattered from atmospheric elements, increases with an increasing target distance. The intensity corresponding to specific objects depends on the distance of the target object from the camera, the radiance of the object, the extinction coefficient of air, and the scattering coefficient of air.

Based on Equation (1), the extinction coefficient (α) can be retrieved if we assume that $C_{1,2}$ does not depend on the direction angle and distance for a given single image. In other words, if we know the pixel values of an arbitrary target, located at different distances in a similar direction and with similar target scattering characteristics, we can retrieve the extinction coefficients. For example, mountains that have similar vegetation and the same angle of inclination as the camera are good candidates. All the types of target objects with the same reflectivity can also be used; however, an object that is closer to black improves the extinction coefficient retrieval because the scattering parameter ($C_{2}$) only contains aerosol information, which dominates Equation (1). As an example, if we have shadow-dark regions in a forest in different mountains, we can assume that $C_{1}\;$is the same for all targets [23,30]. Additionally, $C_{1}\;$is not sensitive to the scattering angle as determined by the sun and camera. For this reason, we assumed that $C_{1}\;$is constant and has no direction dependence when using the shadow regions.

The most important effective variable that determines the value of $C_{2}\;$is the direction angle, which is defined by the three-point sun, target objects, and camera. We cannot assume that $C_{2}\;$is a constant value for every pixel because it depends on the direction cosine between the direction of the sun and the line of the site (direction vector from camera to objects), which have different directions for all the different pixels. In principle, $C_{2}$strongly depends on the scattering phase function, and the scattering phase function depends on the characteristics of the aerosol, such as its size, shape, and refractive index. All of these aerosol characteristics are constant if the target objects are used in similar directions. However, this problem cannot be solved if the adjacent pixels of the image are not selected. This is because the two adjacent pixels have almost the same viewing direction. If two adjacent pixels can be extracted from the image of an object at different distances, $C_{2}\;$can have a constant value.

**Figure 1:**
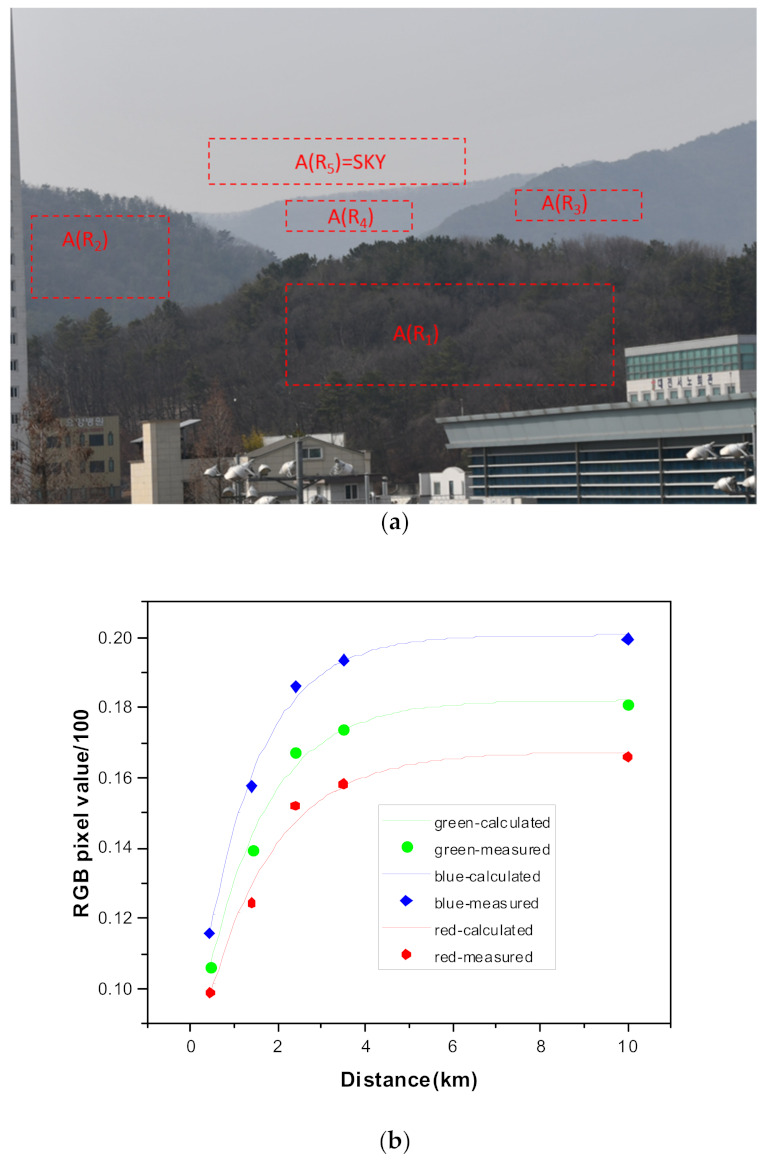
Traditional landscape image and exponential curve shapes when using five different objects, including sky images (438 m, 1.4 km, 2.4 km, 3.5 km, and an assumed 10 km). (**a**) Landscape image and five target objects and (**b**) pixel values and the fitted exponential functions.

However, in reality, as objects at different distances in the same direction cannot be obtained from a single image, we show that extinction coefficients calculated from differently directed target objects do not strongly affect the result within the applicable error range. In most cases, it is possible to use a target object in a similar direction because the systematic error, which depends on the target direction, is within the desired range. To obtain good retrieval conditions by selecting the pixel values $I\left(R_{i}\right)$ of the target objects, we must (1) select an object (mountain) in a similar direction, (2) choose a certain area at the same distance (dashed line area in Figure 1a); A($R_{1}$),…,A($R_{5}$)), and (3) determine the average or minimum value of all pixel values in the selected area.

For the sky, there are no real objects, thus corresponding to an object with zero reflectivity or objects with a small reflectivity, such as shadow objects. This type of sky assumption is equivalent to the fact that the reflectivity of the adopted target object is small and infinitely far. These assumptions can be satisfied irrespective of the type of object when the target object occurs at an infinite distance because the first term of Equation (1) is ignored for long-distance objects despite a high reflectivity.

Figure 1 shows a typical landscape image used in this study. Four subjects and the sky were used in the calculation, whose distances were 0.4, 1.4, 2.4, 3.5, and 10 km (assumed), respectively. In Figure 1, the red-dotted box ($A\left(R_{i}\right)$) indicates the pixel points representing each object; we selected and averaged the small values among the pixels for each value within the given dotted box and used them as the $I\left(R_{i}\right)$ value in Equation (1). Figure 1 compares the pixel values of the target object, including the sky image and the exponential function obtained by the non-linear fitting of these pixel values with the actual value, as shown for the three-color RGB. Figure 1b contains the parameters for the function of Equation (1); the extinction coefficients can be obtained using the selected values and selected pixel values used in the actual calculation.

Effective wavelengths for the RGB wavelengths should be defined more carefully for a broad spectral range of sensors, such as the R, G, and B sensors on a digital camera. We included three parameters: illumination system, sensitivity of the RGB sensors, and scattering efficiency of the aerosols and molecules. If an arbitrary illumination system has a high energy at a given wavelength, this wavelength should be considered as a high-weight wavelength. In contrast, if we have a high sensitivity at specific wavelengths, we should also assign a high-weight value at that wavelength. Finally, if particles have a high scattering efficiency at a specific wavelength, we should also consider this wavelength with a high-weight wavelength. If we consider all of these effects, we can define three RGB effective wavelengths as follows:

(2) $$\lambda_{i}=\frac{\int_{-\infty }^{+\infty }\lambda S_{i}\left(\lambda \right)I\left(\lambda \right)\alpha \left(\lambda \right)d\lambda }{\int_{-\infty }^{+\infty }S_{i}\left(\lambda \right)I\left(\lambda \right)\alpha \left(\lambda \right)d\lambda }\;I=R,G,\;\mathrm{and}\;B$$

where $S_{i}\left(\lambda \right)\;$is the sensitivity of the camera to the R, G, and B sensors, $I\left(\lambda \right)\;$is the illumination light intensity spectrum, which changes with time and region, and $I\left(\lambda \right)\;$ is the extinction efficiency of the aerosols and molecules, which also change with the aerosol characteristics, such as the size, refractive index, and wavelengths [31]. Although the effective wavelengths depend on unknown parameters, such as the Mie scattering effect, we can approximately obtain three effective wavelengths for a given sun light illumination and a given sensitivity characteristic profile ($S_{i}\left(\lambda \right)$). The sensitivity profile was measured using a light source traversing a high-resolution spectrometer, such as a monochromator.

**Figure 2:**
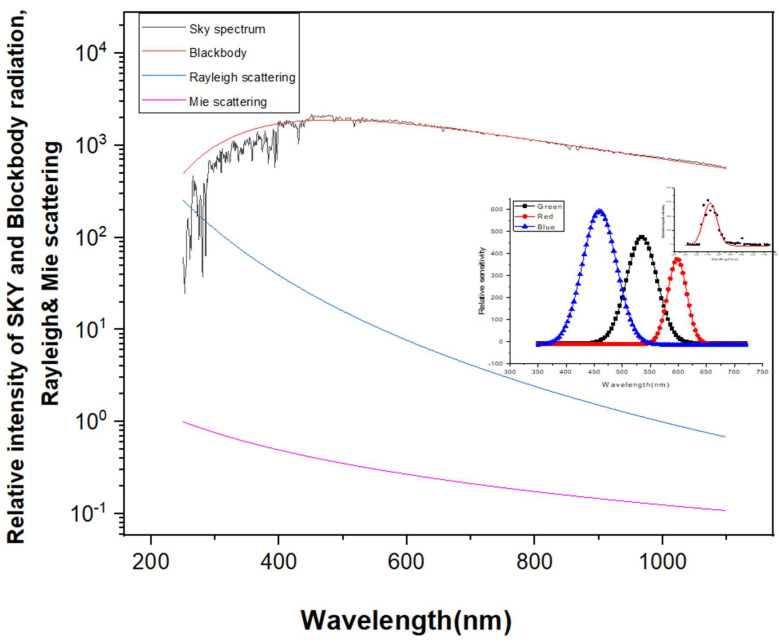
Wavelength-dependent parameters used to calculate the effective wavelengths.

Figure 2 shows the wavelength-dependent parameters, such as the sun scattering illumination spectrum, blackbody radiation, Rayleigh scattering extinction coefficient, and aerosol extinction coefficient. Here, arbitrary units represent the two scattering extinction coefficients, namely, Rayleigh and Mie scattering.

**Figure 3:**
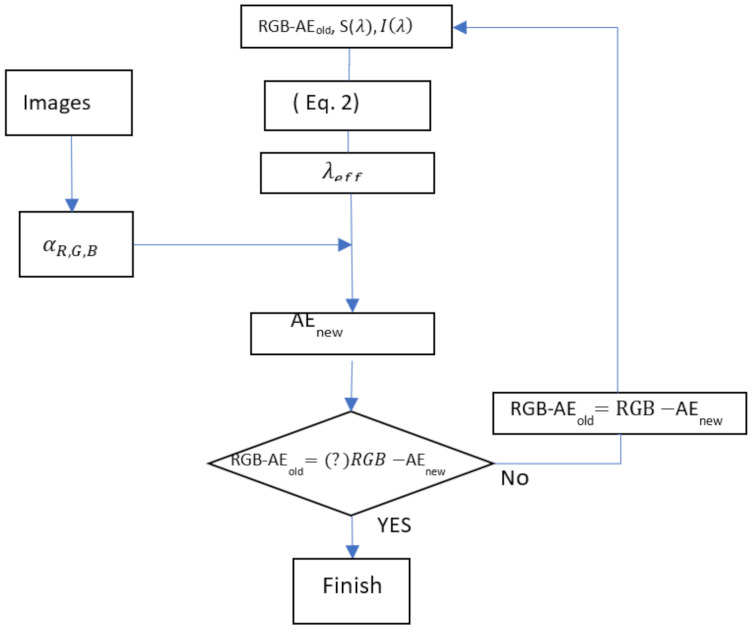
Flow chart for iterative RGB-AE calculation.

In this study, the CCD (Charge-Coupled Device) sensitivity profile was experimentally measured and used in Equation (2); the illumination intensity of sunlight ($I\left(\lambda \right)$) was measured using a spectrometer. At first, we assumed the wavelength dependent extinction coefficients because we had no information on the extinction coefficients at all the wavelengths. The effective wavelengths were calculated by assuming a constant aerosol coefficient. After obtaining the three extinction coefficients ($\alpha \left(\lambda_{i}\right)$) from Equation (1), we obtained the extinction coefficient profile ($\alpha \left(\lambda \right)$) for the full spectral region using RGB-AE (RGB effective wavelength Ångström exponent). Using this extinction profile, the total atmospheric extinction coefficients ($\alpha \left(\lambda \right)$) was applied to Equation (2) to recalculate the effective wavelength ($\lambda_{i},i=R,G,B$). These processes were calculated iteratively until convergence of the effective wavelength and RGB-AE (Figure 3). In this study, RGB-AE was defined to distinguish it from existing conventional AE definitions using extinction coefficients obtained over a wide wavelength range. As defined in Equation (2), the characteristics of the illumination spectrum depend on various variables, such as the direction of the sun, the direction of the camera, the presence or absence of clouds, and the morning and evening directions of the object. As these values vary over time, it is difficult to directly compare the effective wavelength and AE of the dissipation coefficient obtained by this method with conventional values. Here, we note that these RGB-AEs and effective wavelengths can change depending on the aerosol conditions, weather conditions, and illumination conditions, among others.

### 2.2. Dependence of Assumed-Sky Distances

**Figure 4:**
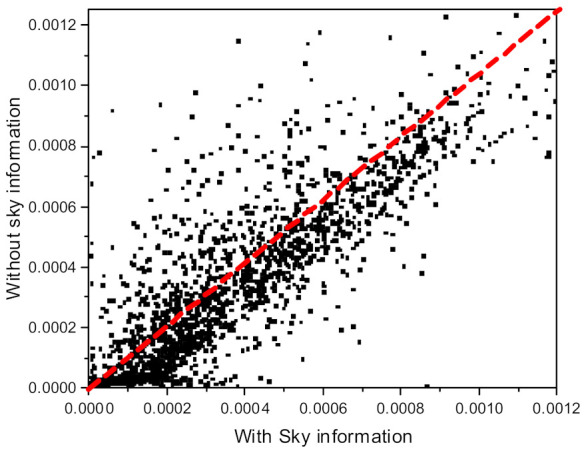
Correlation of the extinction coefficients between two cases. The x-axis represents the extinction coefficients measured with the present far distant sky objects and the y-axis represents the sky image assuming that the sky object is located at a distance of 10 km.

**Figure 5:**
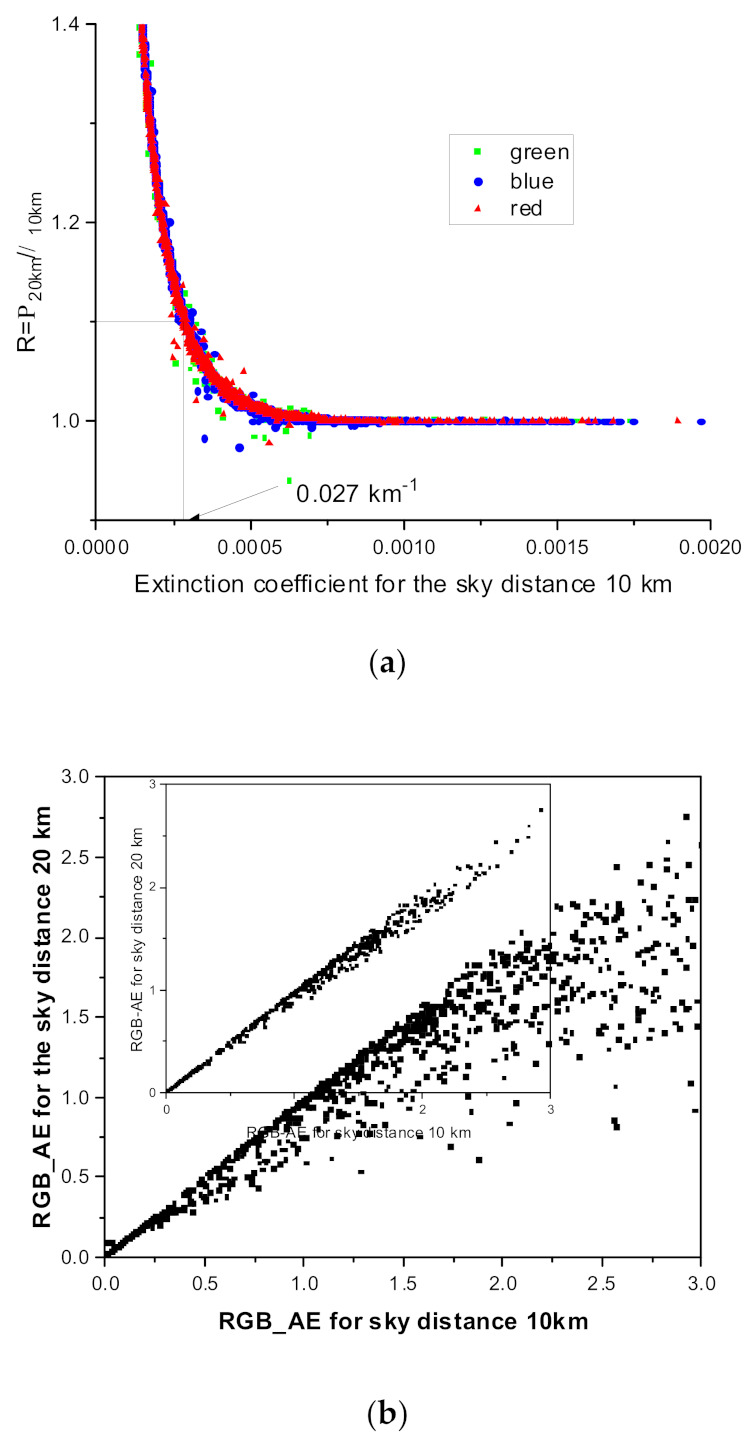
Comparison between two extinction coefficients and the RGB-AE variation calculated using two different sky target distances. (**a**) Comparison between two extinction coefficients calculated using two different sky target distances (10 km and 20 km) at RGB wavelengths. (**b**) Comparison between two RGB-AEs calculated using two different sky target distances for the RGB-AE ranges. The inset shows the same comparison for extinction coefficients ≥ 0.27 km^−1^.

Among the target objects used to inversely calculate the extinction coefficient with Equation (1), the pixel value of the object at an extended distance strongly affects $C_{2}$, as shown in Equation (1). If there is no such distant object, we can introduce the sky as a long-distance object in the calculation. We investigated the retrieval characteristics of the extinction coefficient according to the presence or absence of long-distance objects. Figure 4 shows the correlations between the two methods based on measurements from December 2020 to March 2021. Figure 4 shows the correlation between the extinction coefficients using only four objects (method 1) and the extinction coefficients using additional objects that existed in the sky at a distance of 10 km (method 2). The y-axis represents the extinction coefficients calculated using four objects located in a similar direction (Figure 1a). In contrast, the x-axis represents the extinction coefficients calculated by assuming that additional objects occur 10 km in the sky direction (Figure 1a). As shown in Figure 4, the extinction coefficients calculated without sky images have lower values compared with those calculated with sky pixels. Based on the difference between the two values calculated with and without the sky pixels, a low extinction coefficient yields a large difference between the two methods. Although not presented in Figure 4, for the same reason, the extinction coefficient in red color shows more differences than that in blue for the two retrieval methods owing to the low extinction coefficient in the red color. We can know that these differences can be sufficiently removed by increasing the target object distances used for calculation. We will discuss this in Section 2.5. If we only use days with extinction coefficients > 0.27 km^−1^ (see Figure 5) obtained using approximately 2300 images from December 2020 to March 2021, the correlation coefficients for both the blue and red wavelengths are approximately 0.84.

**Figure 6:**
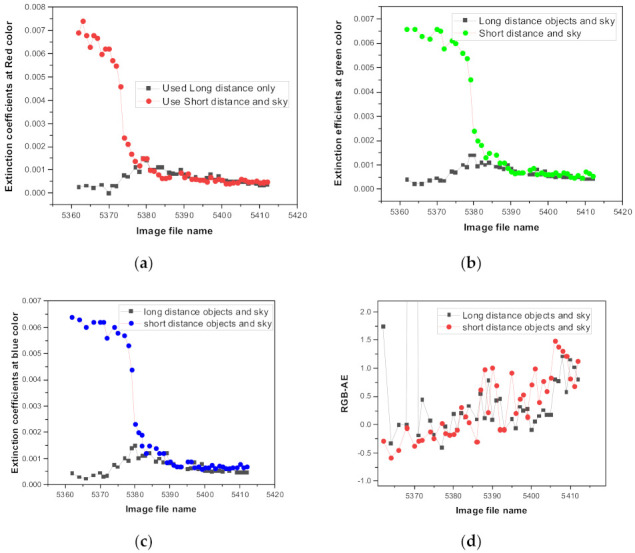
Extinction coefficients and RGB-AE obtained on a foggy day using objects at various distances. (**a**) Extinction coefficient shown in red, (**b**) extinction coefficient in green, (**c**) extinction coefficient in blue, and (**d**) RGB-AE.

To determine the effects of the assumed sky distance on extinction coefficient retrieval, we calculated the extinction coefficients using different sky distances. Figure 5a shows the correlation between the two sky distances assuming that the sky object is at 10 and 20 km. The shorter the sky distance, the smaller the extinction coefficient. This phenomenon is the difference that occurs when the distances between the target objects—the four objects in Figure 1a—are not sufficiently large. Based on Figure 5a, when the extinction coefficient is 0.27 km^−1^, the two methods show a 10% difference. This difference occurs because the distances of the target objects are not sufficiently large, as compared with the visibility. When the visibility is small, we can assume that the extinction coefficient and information on $C_{1}$ and $C_{2}$ do not include the pixel value of the target object within a small distance. Mathematically, when the extinction coefficient is small, the exponential function in Equation (1) can be expressed as a linear function. When the sky at a given brightness is at approximately 10 km, there is an increase in the slope; thus, the pixel value of the sky is not yet saturated, yielding a smaller value. In contrast (20 km), the value is already saturated, indicating a large extinction coefficient. In most countries, meteorological/environmental problems occur at a low visibility. Therefore, the above difference does not pose a significant problem when applied to an actual situation. However, to reduce this error, we recommend using target objects that are as far away as possible. Additionally, based on Figure 6, this phenomenon occurs at the same quantity and scale at all wavelengths owing to the above explanation. In other words, when the extinction coefficient is >0.27 km^−1^, the difference between the two different sky distances is <10%. Therefore, when the extinction coefficient is large, the distance assumption for the sky image or pixel value does not affect the inverse calculation of the extinction coefficient.

Furthermore, based on Figure 5b, the RGB-AEs, which are calculated assuming that the sky image distance is 10 km, have higher values than the other cases. When the constants ($C_{1}$ and $C_{2}$) are known, the extinction coefficient can be expressed in proportion to the reciprocal value of the distance. For this reciprocal dependence with the distance, the RGB-AE value obtained with the sky image at 10 km always has a large value compared with 20 km. However, when the extinction coefficient is >0.27 km^−1^, the variation in the RGB-AE values due to the distance difference decreases as we can more accurately calculate the extinction coefficient. The inset in Figure 6b shows extinction coefficients ≥ 0.27 km^−1^. In conclusion, using distant target objects is optimal on days with good visibility.

### 2.3. Dependence of Object-Distances

From a mathematical perspective, to obtain $C_{1},\;C_{2}$ and α, which define the exponential functions in Equation (1), we must use target objects located at various distances. We tested the characteristics of the retrieved extinction coefficient using different subjects at various distance combinations in a foggy atmosphere when the atmospheric characteristics changed significantly from low to high visibility. Figure 6 shows the characteristics of the retrieved extinction coefficients using different target combinations. Black profiles were retrieved using mountain targets at 1.2 km, 3.6 km, 6.1 km, and sky. In contrast, the colored RGB extinction profiles were retrieved using black building windows located at 50 m, 110 m, 185 m, 270 m, and sky. The x-axis of Figure 6 represents the unique number of photos, where a difference of 1 represents 5 min [32].

In the presence of fog, most distant objects show similar pixel values, such that they do not show any difference in their pixel values depending on the distance; therefore, short-distance target objects only affect the extinction coefficients. If we use distant target objects, we obtain similar pixel values and similar extinction coefficients, as shown in Figure 6. In contrast, using close objects for retrieval, yields more diverse extinction coefficient values. In a thick fog state, the pixel values only change for near-distance objects, but the pixel values become saturated for distant objects. From a mathematical perspective, this is because the pixel value of an object at a short distance, used together with the pixel signal in the sky can properly represent the exponential function given by Equation (1). Therefore, to apply this method to various atmospheric environments, we must use as many objects as possible, which will be at various distances for the same type in a similar direction. Figure 6d shows the RGB-AE, which indicates that the value gradually increases from negative to positive as the fog disappears. Although negative RGB-AE values are not frequently observed under normal atmospheric conditions, they are frequently observed during exceptional atmospheric conditions, such as fog, rain, smog, or large particles [33]. Therefore, on foggy days, the direction indicator light at an airport sometimes appears blue. The volume extinction efficiency has a peak value with respect to the change in the particle size; this volume extinction efficiency decreases when the particle size increases above this peak [31]. The change in the RGB-AE specific value according to the particle size can be used to measure and predict the subsequent increase in the size of the fog particles. These calculations were carried out later, and only qualitative effects were analyzed in this study.

### 2.4. Dependence of Objects-Direction

**Figure 7:**
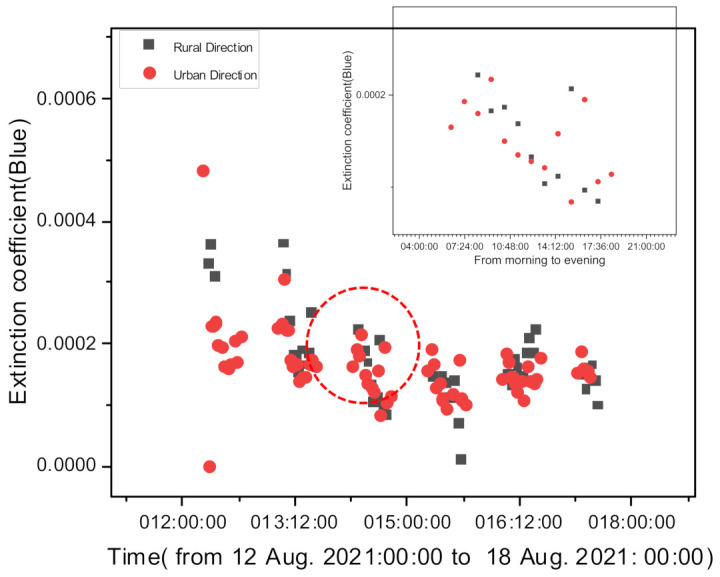
Daily change in the extinction coefficients measured in the downtown direction and the National Park direction in Daejeon, Korea for one week from 12 to 17 August 2021.

**Figure 8:**
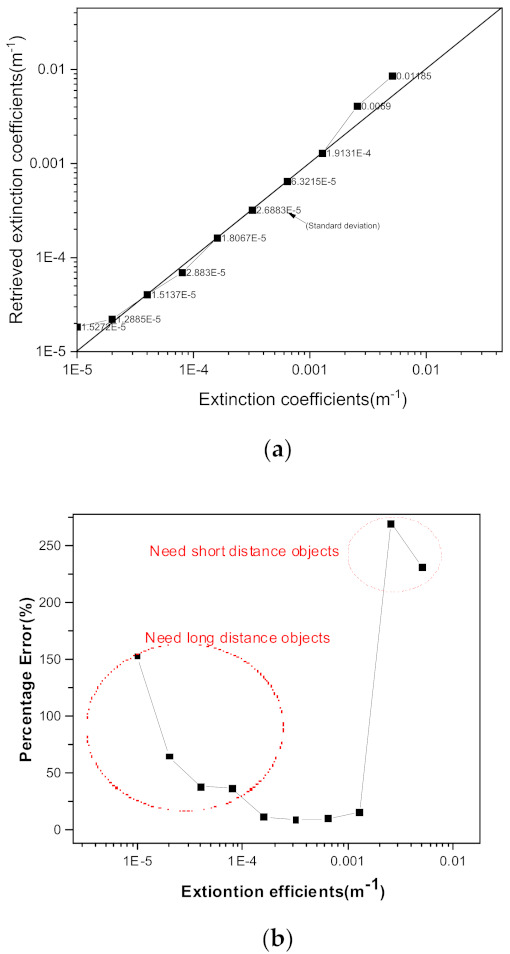
(**a**) The retrieved aerosol extinction coefficients and assumed aerosol extinction coefficients when object-refection and aerosol scattering coefficients change randomly within 10%. (**b**) The percentage errors of the retrieved extinction coefficients for the same target distances (438 m, 1200 m, 2400 m, 3400 m).

Figure 7 shows a comparison of the urban and rural direction (National Park) extinction coefficients for one week at the same site in Daejeon, Korea. Figure 7 verifies whether the direction and distance of target-object images can affect the retrieval of the extinction coefficients using Equation (1). The values of the extinction coefficients obtained in each direction show a similar long-term trend; the response to the occasional rapid large change in the value shows a similar trend. The box graph in Figure 8 shows the extinction coefficients measured on a clean stable day (14 August 2021). To measure the extinction coefficients, we used different target objects with varying directions and distances for the urban and rural cases. As mentioned above, although objects at different distances were used to calculate the extinction coefficients, the calculations yielded similar trends. When we image objects located in a different direction compared with the sun, the scattering of aerosol in that direction can change the value of $C_{2}$ in Equation (1) because the scattering angle is different. However, if scattering does not differ significantly, we can assume that $C_{2}$ is constant in Equation (1), neglecting the variation in the $C_{2}$ value.

### 2.5. Dependence of Target-Reflectance and Particle Scattering Efficiency

Figure 8 shows the correlation between the retrieved extinction coefficients and real-assumed extinction coefficients (a) and percentage errors (b). This figure is simulated and results with the assumption that the reflectivity of the five objects (corresponds to the $C_{1}$s) and the scattering coefficients (correspond to $C_{2}$s by suspended particles) changes randomly within 10% simultaneously. In this study, we assumed that the distances of objects (located at the 438 m, 1200 m, 2400 m, 3400 m) have no measurement error because we can measure distance exactly using lidar or other methods. As Figure 8a,b shows, the optimum aerosol extinction coefficients is around 0.0005 m^−1^ and the applicable aerosol extinction coefficients ranges from $5\times 10^{-5}$ to $1\times 10^{-3}$ m^−1^. In most case the aerosol extinction coefficients are within these range, so we think that the target distance distributions used in this study are reasonable in retrieving everyday weather conditions [34,35]. However, to obtain a wider range of aerosol extinction coefficients, the same objects at different distances must be used. For this reason, the camera needs to be positioned in a suitable position and direction so that it can use different objects, and it can also use different sets of objects (different $C_{1}$ values) at the same time. The use of these different types of objects may be the subject of future research.

## 3. Dependence on Weather Conditions

In this section, the extinction coefficients were obtained under various atmospheric conditions to prove the proposed method while the extinction coefficient behavior was compared with the well-known aerosol scattering theory and other existing aerosol studies.

**Figure 9:**
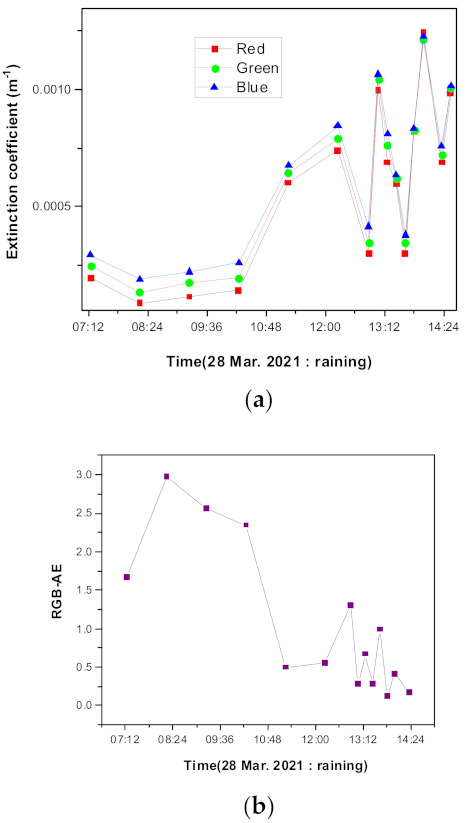
Extinction coefficients and AE characteristics on a rainy day (28 March 2021): (**a**) extinction coefficients and (**b**) RGB-AE.

Figure 9 shows the results measured on a rainy day. The RGB-AE and extinction coefficients change substantially depending on the occurrence of precipitation. Based on these results, we also suggest that the extinction coefficients can be realistically measured using a commercial camera. At the time of image capture, there is no direct sun illumination and the reflection effect can be ignored in these measurements. However, the presence of clouds can change the illumination conditions, effective wavelengths, and RGB-AE. Although the RGB-AE values in Figure 9b cannot be obtained accurately due to the effective wavelength obtained through the assumption of a constant illumination spectrum profile, the extinction coefficient and RGB-AE values obtained at the time of precipitation and when it stops are distinctly different. This value can be used to estimate the presence or absence of rainfall. Figure 9b also shows that when it rains intermittently, the RGB-AE values do not recover the AE to its original state due to strong humidity.

**Figure 10:**
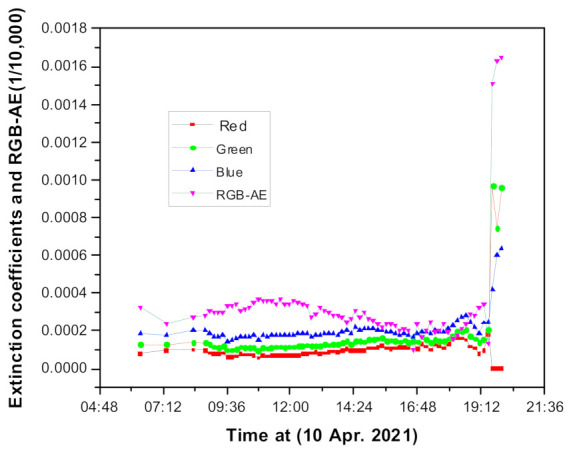
Extinction coefficients and AE value variation measured during the daytime.

**Figure 11:**
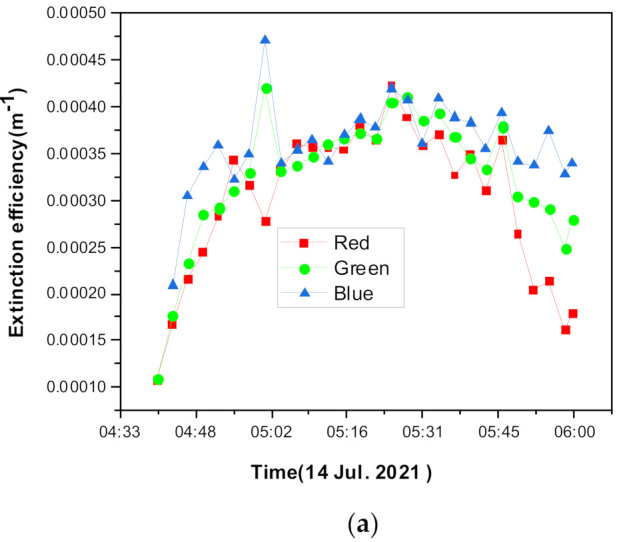

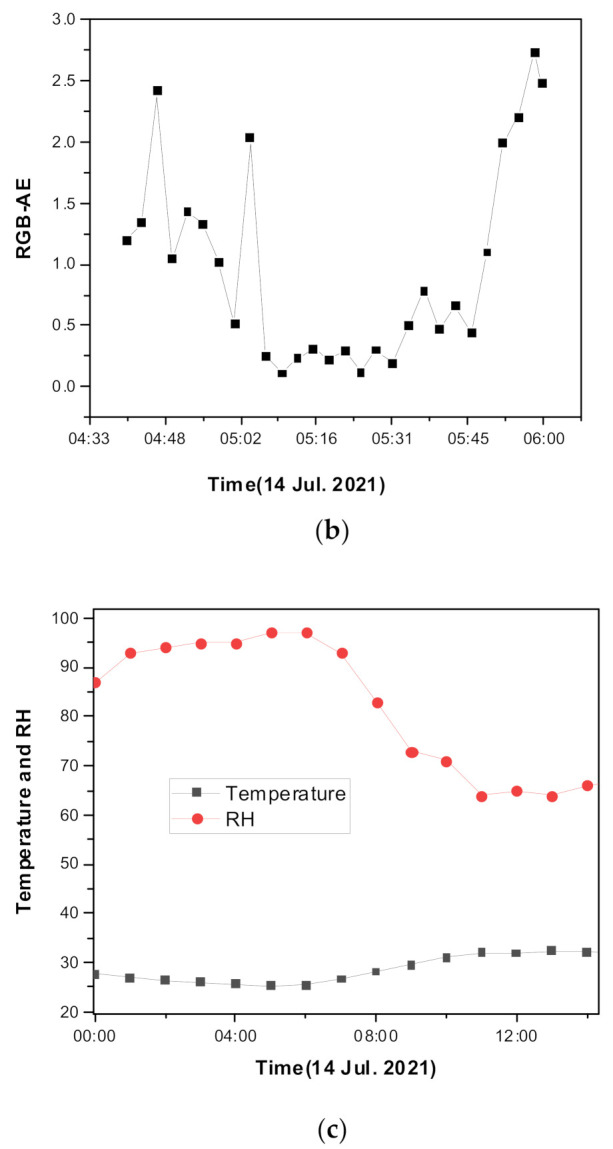
Aerosol extinction and the RGB-AE characteristics based on the changes in the humidity/temperature measured in early morning twilight conditions: (**a**) extinction coefficients, (**b**) RGB-AE, and (**c**) humidity and temperature.

Figure 10 shows the temporal behavior of the extinction coefficient and RGB-AE from early morning to sunset for a fixed camera orientation. Twilight and sunset on this date were 05:39 and 17:25, respectively, on a clear day (10 April 2021). As shown in Figure 10, we can obtain the extinction coefficients and AE values regardless of the position of the sun or relative direction of the camera from sunrise to sunset. Generally, sunlight will pass the long-distance-atmospheric path at sunset or sunrise, such that long wavelengths are dominant in the atmosphere under these conditions; thus, the red-light source acts strongly in aerosol scattering based on Equation (2). Considering this point, the effective wavelength also shifts from the short-wave wavelength to the long-wave wavelength, and the effective wavelength leads to sequential changes in the RGB-AE. We did not measure the time-dependent illumination light intensity profile. In the early morning and late evening, we cannot directly compare the RGB-AE with the conventional AE owing to these changes in the effective wavelength. The value of the extinction coefficient after 19:00 cannot be used because, as shown in Figure 11, sunset completely occurs after 19:00, and these effects cause a significant error in the calculation value. The RGB-AE, which is related to the particle size, shows the largest value during the day and the smallest value near sunset and sunrise. This can be predicted in connection with the change in the humidity, but this daily change does not always appear. More importantly, in Figure 11, we highlight that the relatively low 0.1 km^−1^ extinction coefficient is continuously maintained within the error range of the device.

To verify that the proposed method can be applied for a small amount of light, regardless of the amount of light, changes in direction, changes in light intensity, and changes of humidity, an observation was performed with a high temporal resolution at sunrise. Figure 11 shows the changes in the aerosol extinction and RGB-AE in the early morning on a high relative humidity day. The civil twilight on this day was 04:54.

The extinction coefficient, Figure 11a, continuously increased until sunrise and then decreased again after the sun had risen owing to the influence of the relative humidity. Similarly, the RGB-AE values, Figure 11b, showed a minimum value at the points where the particle size can be considered the largest. After sunrise, this AE value increased up to the normal value, which is expected for a normal aerosol size distribution. These phenomena can be explained by Figure 11c, where the humidity increases until just before sunrise and then decreases again, according to the changes in the temperature.

**Figure 12:**
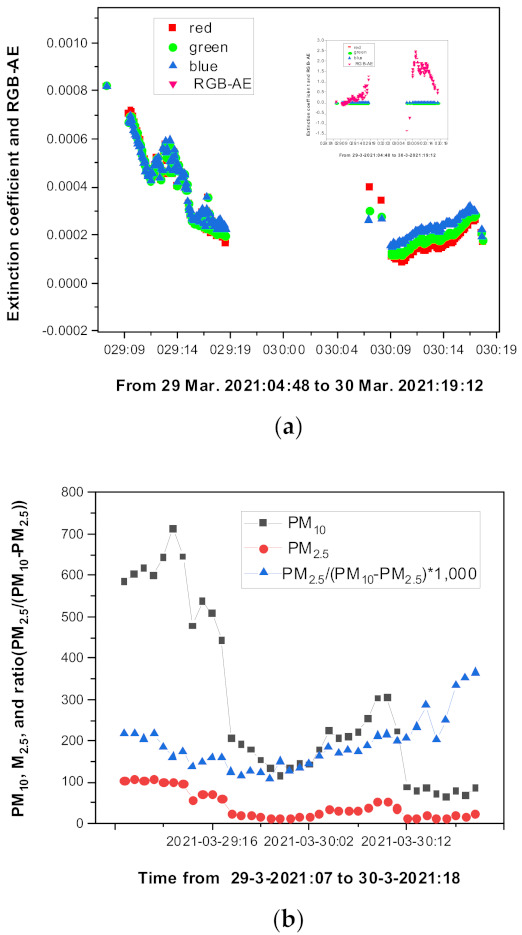
RGB-AE values and extinction coefficients obtained from camera images and PM obtained via the Ministry of Environment Observation network. (**a**) RGB-AE values and extinction coefficients. (**b**) PM_10_, PM_2.5_, and their ratio.

Figure 12 shows the aerosol extinction coefficients and RGB-AE measured using our method on the days when the Asian dust storm occurred in the study area. Night-time data were excluded because it was difficult to measure the extinction coefficient using a camera at night. This measurement was performed on the date when the dust storm was in the process of disappearing from the date of occurrence. From the morning of 29 March to the afternoon of 30 March, strong Asian dust storms occurred, with PM_10_ reaching 700 ${\mu \mathrm{gm}}^{-3}$ on the morning of 29 March. The box plot in Figure 12a shows the RGB-AE, where a more significant density of Asian dust storms results in a smaller RGB-AE value. This phenomenon, which is common for Asian dust storm events, can be easily estimated based on the value of the extinction coefficient at the RGB wavelength, Figure 12a [36,37]. Under normal aerosol conditions, the ratio of fine to coarse particles is approximately 0.6 [38,39,40]; however, for Asian dust storms, based on Figure 12b, the concentration of PM_10_ particles is significantly higher than that of PM_2.5_. In Figure 12b, PM_2.5_/(PM_10_–PM_2.5_) represents the ratio of purely small fine particles to purely large coarse particles. For this change in the size distribution, as shown in Figure 12b, the RGB_AE value is different from that of normal aerosols. Many studies have focused on the change in AE value during an Asian dust storm; our results display the same trend [36].

**Figure 13:**
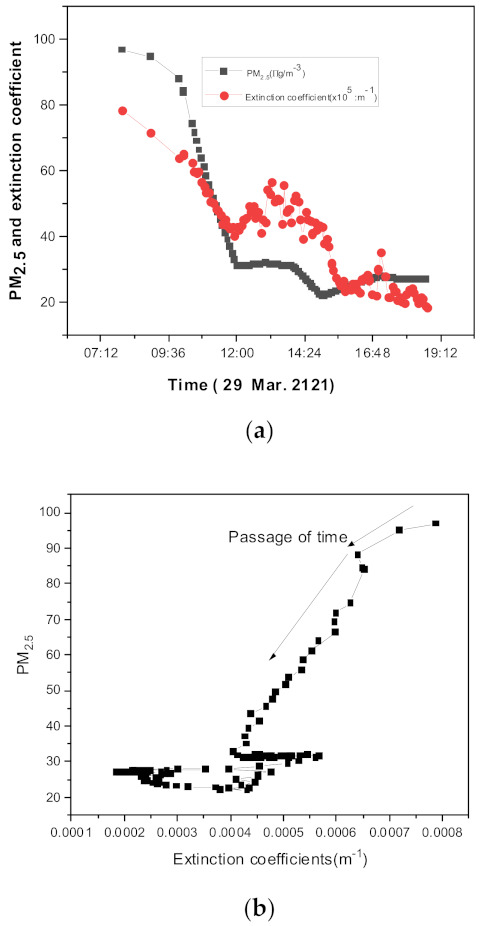
Temporal changes in the extinction coefficients (blue) and PM_2.5_ values obtained on the same date with the disappearance of the dust storm. (**a**) Temporal changes in PM_2.5_ values and extinction coefficients (blue). (**b**) Correlation between PM_2.5_ values and extinction coefficients.

Figure 13 shows the correlation between PM_2.5_ and the extinction coefficient obtained on 29 March when the Asian dust storm occurred in Daejeon, Korea. We used PM_2.5_ for comparison because PM_2.5_ generally has a greater scattering efficiency than PM_10_. Figure 13 shows a part with a strong correlation and a part with a weak correlation. A stronger correlation was observed for strong yellow dust, whereas a weak correlation was observed during the weakened effect of yellow dust. From a theoretical perspective, we cannot have a strong correlation if aerosols derive from varying sources because they have different refractive indices and size distributions [41]. This weak correlation can be explained by the changes in the particle size distributions because the extinction coefficient is the product of the volume extinction efficiency and the volume size distribution product of the particle [31]. The extinction coefficient is more sensitive to the particle size distribution than PM_2.5_, which is an integrated mass within a given size; therefore, we can consider that the particle size distribution changes across a small correlation region.

**Figure 14:**
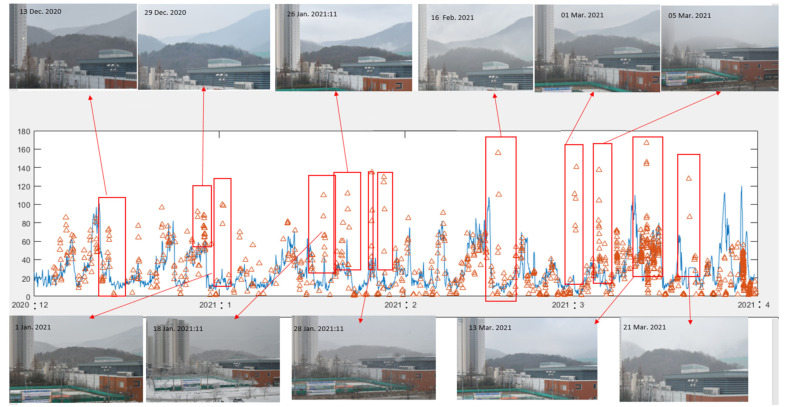
Comparison of the extinction coefficients (blue) measured for four months (from December 2020 to March 2021) and PM_2.5_ values obtained via the domestic environmental measurement network. Photos are shown for the dates characterized by a significant difference between the two values.

Figure 14 compares the extinction coefficient measured using our method with PM_2.5_ values measured by the Korean Ministry of Environment from December 2020 to March 2021. Although the two measurement sites are 3.1 km apart, we can roughly confirm that there is a high correlation between the two measurement values based on long-term observations. However, for some exceptional dates, our measurements yielded high extinction coefficients compared with PM_2.5_ values. Figure 14 provides images for these exceptional dates to determine the reason for the substantial difference. Based on these photos, these special dates had occurrences of fog formation, rain, snow, occasional clouds at the mountain target objects, and occasional thick haze. As mentioned in the Introduction and shown in Figure 13b, the extinction coefficient can have various values even for the same PM_2.5_ value, which depend on the size distribution, relative humidity, and origin of the aerosol. Therefore, it is impossible to directly compare PM_2.5_ values and the extinction coefficients under various weather conditions, such as rain, snow, and fog. In contrast, this discrepancy indicates a change in various meteorological phenomena, such that it can be utilized in other applications.

## 4. Conclusions

We proposed and discussed a new method for measuring the extinction coefficients of atmospheric particulates using an arbitrary landscape image. Considering the spatial and temporal characteristics of aerosols, this method can be effectively applied in various fields because it can rapidly and widely obtain the average value of aerosol extinction, coefficients for large visible areas. The following represent the advantages of this method, (1) extinction coefficients can be obtained regardless of cloud or weather conditions, (2) extinction coefficients can be measured not just at one point, but at an average of points over a wide area, (3) we have no limit in temporal resolution because it depends on the shooting time of the camera, (4) the extinction coefficient can be obtained at three wavelengths at once, the size information of the aerosol can be obtained, (5) most importantly, it provides different physical quantities and units from conventional methods (PM2.5/10 values), (6) the use of the extinction coefficient in conjunction with PM2.5/10 is advantageous to obtain other information about the aerosol (size, density, refractive index).

In Section 2, we discussed the effects of experimental conditions on the retrieving extinction coefficients. We checked the effects of object distances and direction of the targets on the retrieving extinction coefficients and found that the direction of the sun and camera have no effect on the retrieving results. In addition, the characteristics of the retrieved extinction coefficient with the assumed distance of the sky image were analyzed. When the extinction coefficient was higher than 0.27 km^−1^ it was found that the assumed sky distance was not important. Perhaps this extinction coefficient (2.7 km^−1^) depends on the distance of the object used; if we used more far-long distant objects, we think that the sky image distance is not important even at a lower extinction value. In Section 2.5 we measured the dependence of the retrieved extinction when the target reflectance and scattering efficient change (effects of $C_{1}$ and $C_{2}$) randomly when the target distances are measured precisely. We found that target objects distances, 0.4 km, 1.4 km, 2.4 km, and 3.5 km are good candidates for retrieving aerosol extinction coefficients from ranges $5\times 10^{-5}$ to $1\times 10^{-3}$ m^−1^ extinction coefficients.

In Section 3, the extinction coefficient was measured under various atmospheric conditions at three effective wavelengths, as defined by Equation (2). Using these three effective RGB wavelengths, we defined the RGB-AE, which was used to characterize changes in the aerosol characteristics. To show the theoretical reliability of this method, extinction coefficients were calculated for various target object states, with an analysis of their characteristics. As a result of this reliability evaluation, we found that a reliable extinction coefficient can be obtained for a general landscape composed of a mountain background. By measuring the extinction coefficients during special scenarios, such as fog, rain, haze, and yellow sand, our method remained valid. By calculating the RGB-AE under special weather conditions (fog, rain, and snow) or environmental phenomena (such as the Asian dust storm), we found that these values qualitatively represent the particle size, similar to traditional AE values measured by other people. Even though the extinction coefficient and PM_2.5_ values are not the same physical quantity (different unit), long-term observations showed that this method is valuable as a new independent measurement tool. That is to say, even though Figure 14 shows good correlation with PM_2.5_, sometimes it shows different behavior. Finally, as the extinction coefficients represent different aerosol physical parameters, they can be applied to different and independent new fields if we use these methods for extended periods and at a high spatial resolution.

In the future, we will use this method to obtain three longer-term effective wave extinction coefficients. We will also use these two different independent types of information (extinction coefficient and PM_10/2.5_) to obtain more detailed information such as the specific size and chemical-physical properties of the aerosol.

## Data Availability

Not applicable.
